# Protein Disulfide Isomerase Endoplasmic Reticulum Protein 57 (ERp57) is Protective Against ALS-Associated Mutant TDP-43 in Neuronal Cells

**DOI:** 10.1007/s12017-024-08787-0

**Published:** 2024-06-11

**Authors:** Sonam Parakh, Emma R. Perri, Marta Vidal, Zeinab Takalloo, Cyril J. Jagaraj, Prachi Mehta, Shu Yang, Colleen J. Thomas, Ian P. Blair, Yuning Hong, Julie D. Atkin

**Affiliations:** 1 Motor Neuron Disease Research Centre, Macquarie Medical School, Faculty of Medicine, Health and Human Sciences, Macquarie University, Sydney, 2109 Australia; 2 Department of Microbiology, Anatomy, Physiology and Pharmacology, School of Agriculture, Biomedicine and Environment, La Trobe University, Melbourne, VIC 3086 Australia; 3 Centre for Cardiovascular Biology and Disease Research, La Trobe University, Melbourne, VIC 3086 Australia; 4 La Trobe Institute for Molecular Science, La Trobe University, Melbourne, VIC 3086 Australia

**Keywords:** ALS—Amyotrophic lateral sclerosis, PDI—Protein disulphide isomerase, ERp57—Endoplasmic reticulum protein 57, TDP-43 pathology, ER stress

## Abstract

Amyotrophic Lateral Sclerosis (ALS) is a severe neurodegenerative disease affecting motor neurons. Pathological forms of Tar-DNA binding protein-43 (TDP-43), involving its mislocalisation to the cytoplasm and the formation of misfolded inclusions, are present in almost all ALS cases (97%), and ~ 50% cases of the related condition, frontotemporal dementia (FTD), highlighting its importance in neurodegeneration. Previous studies have shown that endoplasmic reticulum protein 57 (ERp57), a member of the protein disulphide isomerase (PDI) family of redox chaperones, is protective against ALS-linked mutant superoxide dismutase (SOD1) in neuronal cells and transgenic SOD1^G93A^ mouse models. However, it remains unclear whether ERp57 is protective against pathological TDP-43 in ALS. Here, we demonstrate that ERp57 is protective against key features of TDP-43 pathology in neuronal cells. ERp57 inhibited the mislocalisation of TDP-43^M337V^ from the nucleus to the cytoplasm. In addition, ERp57 inhibited the number of inclusions formed by ALS-associated variant TDP-43^M337V^ and reduced the size of these inclusions. ERp57 was also protective against ER stress and induction of apoptosis. Furthermore, ERp57 modulated the steady-state expression levels of TDP-43. This study therefore demonstrates a novel mechanism of action of ERp57 in ALS. It also implies that ERp57 may have potential as a novel therapeutic target to prevent the TDP-43 pathology associated with neurodegeneration.

**Supplementary Information:**

The online version contains supplementary material available at 10.1007/s12017-024-08787-0.

## Introduction

Amyotrophic lateral sclerosis (ALS) is an aggressive neurodegenerative disorder affecting motor neurons that overlaps significantly with frontotemporal dementia (FTD). Pathophysiological mechanisms associated with ALS include redox dysfunction, defects in RNA metabolism, induction of both oxidative and endoplasmic reticulum (ER) stress, DNA damage, protein misfolding and induction of cell death (Mead et al., 2023). Most cases arise sporadically (90%) but genetic variations are present in familial forms, including in the superoxide dismutase 1 (SOD1) gene (20% of cases). Variants of Tar-DNA binding protein-43 (TDP-43) are also present in 4–5% of familial ALS patients. Interestingly, the major hallmark of sporadic ALS is the presence of pathological forms of TDP-43 in almost all (97%) ALS and ~ 50% of FTD patients (Mackenzie et al., 2007; Maekawa et al., 2009). TDP-43 pathology is present in motor neurons and glia in the primary motor cortex, corticospinal tract, brainstem, and spinal cord of ALS patients (Jo et al., 2020). Moreover, TDP-43 pathology is also observed in other neurodegenerative disorders such as Lewy body disease (LBD), corticobasal degeneration (CBD), progressive supranuclear palsy (PSP), Huntingtin’s disease (Acewicz et al., 2022) and up to 57% of Alzheimer’s disease (AD) cases (Meneses et al., 2021). Hence TDP-43 pathology is central to neurodegeneration.

Under normal physiological conditions, TDP-43 is localised primarily in the nucleus where it functions in RNA metabolism and DNA repair. However, pathological forms of TDP-43 mislocalise to the cytoplasm, where they aggregate and form inclusions. The abnormal aggregation of ALS-associated mutant TDP-43 involves the formation of non-native disulphide bonds (Cohen et al., 2012). Both loss of its normal role in the nucleus and gain of toxic functions in the cytoplasm are implicated in neurodegeneration (Klim et al., 2021; Wood et al., 2021). Hence, approaches that target TDP-43 pathology and prevent its misfolding and cytoplasmic mislocalisation may be relevant therapeutically in ALS, FTD, AD and other neurodegenerative diseases.

Protein disulphide isomerases (PDI) have been proposed as potential therapeutic targets for neurodegenerative diseases (Perri et al., 2017). The PDI family consists of more than 21 members that contain one or more thioredoxin-like domains that mediate protein folding via both chaperone and oxidoreductase activity. The latter function involves the isomerisation, oxidation and reduction of protein disulphide bonds (Robinson et al., 2023). Endoplasmic reticulum protein 57 (ERp57/PDIA3) is an important family member because it is the closest homologue to protein disulphide isomerase (PDIA1, known as PDI), the prototype of the PDI family, and together PDI and ERp57 mediate disulphide bond formation of most proteins (C. E. Jessop et al., 2009). ERp57 is distinct from PDI because it displays differences in redox potential and substrate specificity (Maattanen et al., 2006). Also, ERp57, but not PDI, is part of a specialised folding complex containing ER chaperones calnexin and calreticulin that facilitates folding of glycoproteins (Catherine E Jessop et al., 2007).

PDI and ERp57 display neuron-specific functions in neurite outgrowth, synaptic function, and neuronal connectivity (Bargsted et al., 2016; Bilches Medinas et al., 2022; Woehlbier et al., 2016). Both exonic and intronic variants in the genes encoding PDIA1 and ERp57 have been identified in ALS patients as possible risk factors (Gonzalez-Perez et al., 2015; Woehlbier et al., 2016). We previously showed that PDI is protective against key phenotypes associated with ALS. PDI inhibited the formation of inclusions, ER stress, ER-Golgi transport defects and apoptosis in neuronal cells expressing either mutant SOD1, TDP-43 or FUS (Jeon et al., 2014; Parakh et al., 2021; Parakh et al., 2020; A. K. Walker et al., 2010). PDI also inhibits the mislocalisation of mutant TDP-43 to the cytoplasm and nuclear import defects induced by mutant FUS in neuronal cells (Parakh et al., 2020, 2021). Transient expression of PDI also improves motor function in zebrafish expressing mutant SOD1 (Parakh et al., 2020). In contrast, the protective functions of ERp57 in ALS are not well studied and have only been described in relation to SOD1. We previously showed that ERp57 inhibits mutant SOD1 inclusion formation and apoptosis in neuronal cells (Parakh et al., 2018). More recently, transgenic expression of ERp57 in the SOD1^G93A^ mouse model improved muscle denervation at early symptomatic stages, and SOD1 aggregation at late disease stages (Rozas et al., 2021). However, it has not been previously examined whether ERp57 is protective against TDP-43 pathology in ALS.

In this study, we show that ERp57 is protective against key features of TDP-43 pathology; mislocalisation to the cytoplasm, inclusion formation, ER stress and apoptosis, in cells expressing ALS-associated mutant TDP-43^M337V^. Furthermore, co-expression of ERp57 with mutant TDP-43^M337V^ led to the formation of significantly smaller inclusions, implying that TDP-43 is a client of the ERp57-folding pathway. These results therefore identify a novel protective role of ERp57 in ALS, implying that it may have potential as a therapeutic target to inhibit TDP-43 pathology associated with neurodegeneration. They also offer novel insights into the protective features of PDI family members in neurodegenerative diseases.

## Materials and Methods

### Cell Lines

Mouse Neuro-2a neuroblastoma cell lines (CellBank, Australia, authenticated by Short Tandem Repeat (STR) analysis) were grown in Dulbecco’s Modified Eagle Medium (DMEM) with 10% fetal calf serum (FCS) incubated at 37 °C with 5% CO_2_.

### Constructs

A construct encoding ERp57 in pcDNA3.1( +) was generously provided by Dr Neil Bulleid, University of Glasgow, UK (C. E. Jessop et al., 2009). Turbo-GFP-tagged TDP-43 wild-type and mutant TDP-43^M337V^ in pCMV6-AC-GFP (Farrawell et al., 2015), and mCherry-tagged TDP-43 wild-type and Q331K mutant in pmCherry-N1 (Walker et al., 2013), were as previously described.

### Transfection Protocol

Lipofectamine-2000 (Invitrogen) was used for transient transfection according to the manufacturer’s protocol. Briefly, a day after plating the cells, 50 μl of Opti-MEM media was mixed with 1 μg of the required plasmid DNA and 51 μl of transfection mix (1:50) Lipofectamine-2000 in Opti-MEM followed by 20 min incubation. Cells were co-transfected with TDP-43 and ERp57 or empty vector and examined at 18 h (Experiment S2) or 72 h post transfection using fluorescence microscopy, unless stated otherwise.

### Immunocytochemistry and Microscopy

Neuronal cells (Neuro-2a) were fixed in 4% paraformaldehyde (PFA), following permeabilization with PBST (0.1% Triton-X) and blocking with 3% bovine albumin serum (BSA) in PBS, followed by incubation with mouse anti-V5 (Abcam), mouse anti-CHOP (Santa Cruz), anti-rabbit cleaved Caspase-3 (Asp 175), (Cell Signalling) antibodies in PBS at 4 °C overnight. Secondary antibodies, AlexaFluor 488 or 555 conjugated rabbit anti-mouse IgG (1:250), AlexaFluor 594 conjugated goat anti-rabbit IgG (1:250), were added for 1 h and incubated in the dark at room temperature. After washing with PBS, Hoechst 33342 dye (Invitrogen) was used to stain the nucleus. Photomultiplier sensitivities and offsets in dual-channel imaging were tuned to a level where impacts from bleed-through from one channel to the other were minimal. In all experiments, the gain values of all channels remained consistent. Cells expressing TDP-43 with empty vector or TDP-43 with ERp57-V5 were selected using the drawing/selection tool (freeform, Image J) to quantify fluorescence intensity, according to the formula CTCF = Integrated Density—(Area of Selected Cell Mean Fluorescence of Background Readings). Set measures was chosen from the ‘Analyse’ menu by marking AREA, INTEGRATED DENSITY, and MEAN GREY VALUE. By choosing “Measure” from the ‘Analyse’ menu, the cell fluorescence was measured first in the nucleus and then throughout the entire cell. Background was defined as the region surrounding the cell that lacked fluorescence. The size of inclusions and co-localisation were quantified using ‘3D objects counter’ plugin, ImageJ software.

### Protein Unfolding Assay

Neuro-2a cells were rinsed with PBS and then treated with freshly diluted TPE-MI dye (50 μM in PBS) for 30 min at 37ºC. Excess dye was removed by washing with PBS. Images were obtained using a high-end LSM 880 Zeiss microscope. TPE-MI: excitation: 355 nm, emission: 445–500 nm, DRAQ5tm fluorescent probe which emits in the far-red region, was used as nuclear stain.

### Filter-Trap Assay

Cell lysates were collected by adding chilled Tris-NaCl buffer (50 mM Tris–HCl pH 7.5 and 150 mM NaCl, pH 7.6) with 0.1% [w/v] sodium dodecyl sulfate (SDS), 1% Triton-X100, 1% protease inhibitor cocktail (Roche) and 1% phosphatase inhibitor (Roche) following incubation on ice for 20 min, then stored at -20 °C overnight. To obtain the SDS-soluble fraction, samples were centrifuged at 100,000 g at 4 °C for 30 min and a BCA protein assay was performed (Thermofisher Scientific). The insoluble lysates were prepared by adding 8 M Urea to the pellet, which was then solubilised by sonication and collected following centrifugation. Dot blotting was performed using Bio-Dot Microfiltration Apparatus (Bio-Rad, cat. No. 170–6545) under vacuum. The nitrocellulose membrane was pre-wet in TBS for 10 min before applying to the apparatus, and 100 µl TBS was added to rehydrate the membrane. Insoluble (20 µl) and soluble (20 µg) protein lysates were added to each well and after 40 min, they were washed with TBS before staining with Ponceau S was performed to detect total protein with Chemidoc (Bio-Rad). The membranes were then cut and incubated with 3% BSA (before probing with anti-phospho-TDP-43 antibody) or 5% skim milk (before probing with anti-TDP-43 antibody) for 1 h at room temperature. The membrane was incubated with primary antibodies, anti-phospho-TDP-43 (1:1000, Cosmo Bio Co, TIP-PTD-P05) or anti-TDP-43 antibody (1:1000, ProteinTech, 10,782–2-AP), at 4 °C for 24 h. Following incubation with secondary antibody (1:2500, HRP-conjugated goat anti-rabbit Merck Milipore (AP132)) for 1 h at room temperature membranes were treated with Clarity™ Western ECL Substrate (Bio-Rad).

### Statistics

Experiments were performed three times independently (except the filter trap assay, which was performed twice), with at least one replicate performed blinded. In total, 80–150 cells were examined per group, except for the unfolding assay (Fig. 4A), when a total of 25 cells per group were quantified. Statistical analyses were performed using ANOVA followed by Tukey’s multiple comparison test for all experiments except Fig. 3C and F where a Welsh t-test was performed (GraphPad Prism 5, San Diego, CA, USA). P-values of 0.05 or less were considered significant, *p < 0.05, **p < 0.01, ***p < 0.001, **** p < 0.0001, mean ± SD.

## Results

### ERp57 Reduces the Steady-State Levels of TDP-43 at Low Levels of Expression

We first examined whether ERp57 is protective against TDP-43 pathology induced by the p.M337V ALS-variant in neuronal cells. TDP-43^M337V^ was selected because it has a long half-life and ALS patients bearing this variant display early disease onset (Corcia et al., 2012; Watanabe et al., 2013). Moreover, in neuronal cells, TDP-43^M337V^ mislocalises to the cytoplasm, forms inclusions and induces apoptosis (Parakh et al., 2020). Neuro-2a cells were co-transfected with either wild-type TDP-43 or TDP-43^M337V^, both tagged with Turbo-GFP, and either ERp57, tagged with V5, or empty pcDNA3.1 vector as a control. Immunocytochemistry was performed using an anti-V5 antibody to detect ERp57 expression, and individual transfected cells were examined 72 h post transfection using confocal fluorescent microscopy. ERp57 was found to be co-expressed with TDP-43 in almost 99% of transfected cells (middle panel, Supplementary Figure S1A).

The steady-state expression levels of both ERp57 and TDP-43 in transfected cells were then examined. Western blotting revealed that similar levels of ERp57 were present amongst the groups, and equivalent levels of wild-type and mutant TDP-43 were expressed in the absence of ERp57 (Figure S1B). Surprisingly, however, the steady-state levels of both wild-type and mutant TDP-43^M337V^ were reduced when co-expressed with ERp57 (Figure S1B). This was examined further using confocal fluorescent microscopy. TDP-43 GFP fluorescence pixel intensities of individual cells were quantified using ImageJ. Cells expressing TDP-43 displayed variable fluorescence intensities, and thus levels of TDP-43, which were categorized into low intensity (TDP-43 fluorescent pixel intensity 1000–3000) and mid-higher intensity (TDP-43- fluorescent intensity 3000–9000). Over-saturated cells, defined specifically as those with TDP-43-pixel fluorescent intensity of more than 10,000 (high intensity), were excluded from all analysis because this leads to mis-representation of the actual fluorescence within the cells (Schmied et al., 2023). Consistent with the western blotting results, at the lower range of TDP-43 expression (< 3000, TDP-43 fluorescence intensity), significantly less TDP-43 was detected in cells co-expressing ERp57 compared to those co-expressing empty vector (*p < 0.05), for both wild-type and mutant TDP-43^M337V^ (Supplementary Figure S1C). This finding is consistent with previous observations that ERp57 regulates the steady-state levels of other misfolded proteins associated with neurodegeneration (Torres et al., 2015). However, at the mid-higher range of TDP-43 expression (TDP-43 fluorescent intensity > 3000), no significant differences in TDP-43 levels were detected amongst the groups (Supplementary Figure S1D). Hence, to investigate whether ERp57 is protective against TDP-43 pathology, for all subsequent experiments, only transfected cells with steady-state levels of TDP-43 > 3000 TDP-43 fluorescent intensity were examined, because ERp57 does not modify expression of TDP-43 in this range. Hence it could be ascertained that similar levels of TDP-43 were expressed in the ERp57 co-expressing cells compared to the equivalent group with empty vector. Furthermore, the levels of TDP-43 are directly proportional to its toxicity in neurons, and increased levels of TDP-43 correlate with more severe neurodegeneration (Barmada et al., 2014). Thus, cells expressing the mid-higher range of TDP-43 allow the relevant phenotypes to be monitored. Therefore, we examined individual transfected cells specifically within this range only in subsequent experiments, to examine the effect of ERp57 on mutant TDP-43 induced cellular defects.

### ERp57 Inhibits the Cytoplasmic Mislocalisation of Mutant TDP-43 in Neuronal Cell Lines

**Fig. 1:**
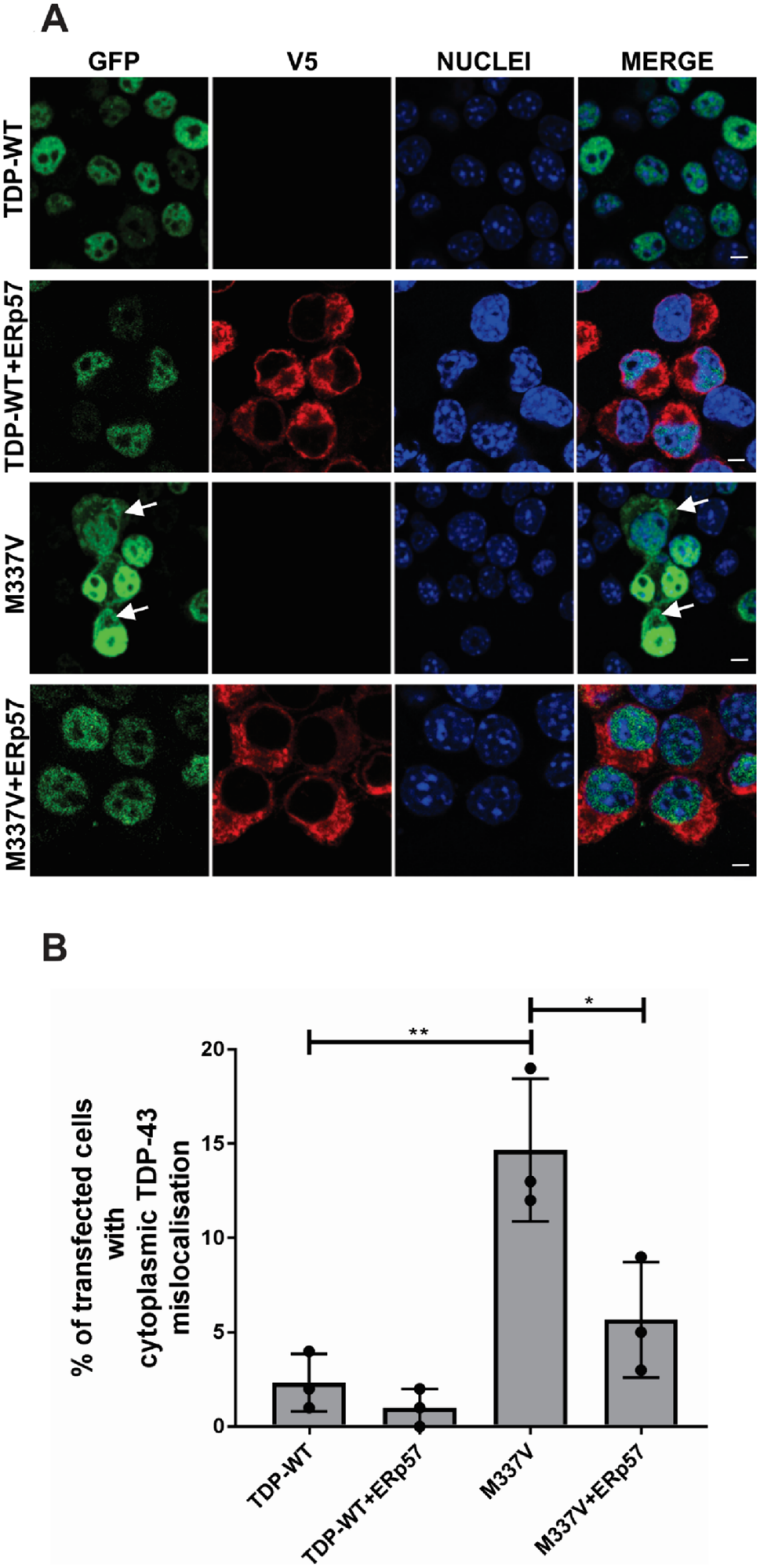
ERp57 protects against mislocalisation of mutant TDP-43 from the nucleus into the cytoplasm in neuronal cell lines **A** Immunocytochemistry and confocal microscopy of mislocalised cytoplasmic TDP-43 in Neuro-2a cells co-expressing TDP-43 and ERp57, at 72 h post transfection. Cells expressing Turbo-GFP tagged wild-type TDP-43 (TDP-WT, panel 1) with pcDNA3.1 empty vector or wild-type TDP-43 co-expressing ERp57 (panel 2) displayed mainly nuclear TDP-43 localisation, whereas more cells expressing mutant TDP-43^M337V^(M337V) exhibited cytoplasmic distribution, indicated with white arrows (panel 3). On co-expressing ERp57 with mutant TDP-43^M337V^, (panel 4) fewer cells displayed cytoplasmic TDP-43 compared to those expressing TDP-43^M337V^ with vector alone. White arrows represent TDP-43 expression in the cytoplasm. Scale bar = 5 µm. **B** Quantification of cells in 1A, displaying cytoplasmic distribution of TDP-43. Results are expressed as mean ± SD, n = 3. Significant differences in the proportion of cells with cytoplasmic TDP-43 were detected between wild-type TDP-43 and mutant TDP-43^M337V^ (**p < 0.01). Over-expression of ERp57 with mutant TDP-43^M337V^ significantly decreased the proportion of cells displaying cytoplasmic TDP-43, compared to TDP-43^M337V^ cells expressing empty vector only (*p < 0.05)

A key feature of TDP-43 pathology is the accumulation of TDP-43 in the cytoplasm (Barmada et al., 2010). Hence, this was next examined in ERp57 and TDP-43^M337V^ co-expressing cells (Fig. 1A) with steady-state levels of TDP-43 > 3000 total corrected fluorescence. Only 2% of cells expressing wild-type TDP-43 displayed cytoplasmic mislocalisation, and this proportion did not significantly change on co-expression with ERp57 (1%). Significantly more cells expressing TDP-43^M337V^ cells displayed cytoplasmic TDP-43, as expected (15%, **p < 0.01). Moreover, when ERp57 was co-expressed with mutant TDP-43^M337V^, significantly fewer cells displayed cytoplasmic mislocalisation compared to those expressing empty vector (6%, *p < 0.05, Fig. 1B). Hence these findings reveal that ERp57 prevents the cytoplasmic mislocalisation of TDP-43^M337V^.

To validate these results, we also examined a second commonly occurring missense ALS-linked mutant, TDP-43^Q331K^, which was tagged with mCherry rather than Turbo-GFP, to confirm that the results obtained were independent of the fluorescent tag. Neuro-2a cells were co-transfected with mCherry tagged wild-type TDP-43 or mutant TDP-43^Q331K^, with either ERp57-V5 or empty vector (Figure S2A). Only 10% of cells expressing mCherry-tagged wild-type TDP-43 displayed cytoplasmic mislocalisation of TDP-43, and this proportion was not significantly altered following co-expression with ERp57 (6%). Significantly more cells expressing mutant TDP-43^Q331K^ displayed cytoplasmic mislocalisation as expected (18%, *p < 0.05). However, when ERp57 was co-expressed with TDP-43^Q331K^, significantly fewer cells displayed cytoplasmic mislocalisation compared to cells expressing TDP-43^Q331K^ with empty vector (10%, *p < 0.05, Figure S2B). Hence, ERp57 inhibits the cytoplasmic distribution of two ALS-linked TDP-43 variants in neuronal cells.

### ERp57 Inhibits the Formation of Mutant TDP-43 Inclusions in Neuronal Cells

**Fig. 2:**
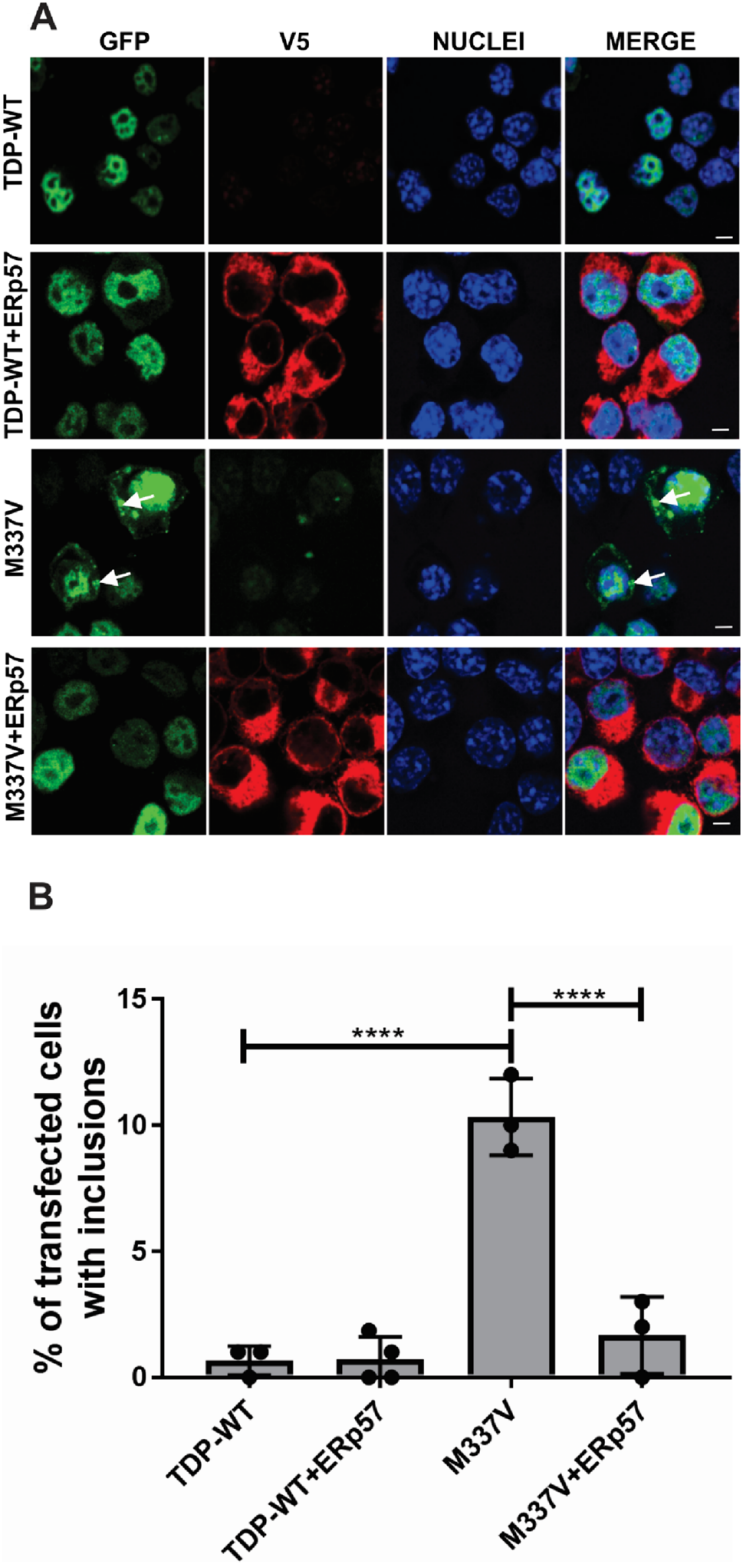
ERp57 inhibits the formation of mutant TDP-43^M337V^ inclusions in neuronal cell lines **A** Immunocytochemistry and confocal microscopy of GFP-positive inclusions present in Neuro-2a cells expressing wild-type TDP-43 (TDP-WT) or mutant TDP-43^M337V^ (M337V, green) with V5 tagged ERp57 (red), at 72 h post transfection. Nuclei are visualised by Hoechst staining (blue). A small proportion of cells expressing wild-type TDP-43 formed inclusions (panel 1), but this was not altered by co-expression with ERp57 (panel 2). More inclusion-positive cells were present in populations expressing mutant TDP-43^M337V^ with vector alone (panel 3) represented by white arrows. In contrast, fewer cells formed inclusions when ERp57 was co-expressed with mutant TDP-43^M337V^ (panel 4). White arrows represent TDP-43 inclusions in the cytoplasm. Scale bar = 5 µm. **B** Quantification of the percentage of transfected Neuro-2a cells bearing inclusions represented in 2A. Results are expressed as mean ± SD, n = 3. Significant fewer TDP-43 inclusions were present in wild-type TDP-43 compared to mutant TDP-43^M337V^ populations (****p < 0.0001). Significantly fewer cells formed inclusions when ERp57 was co-expressed with TDP-43^M337V^ (****p < 0.0001) compared with cells transfected with pcDNA3.1 empty vector alone

Next the formation of TDP-43 inclusions was examined in individual cells co-expressing ERp57 and TDP-43, specifically within  the range > 3000 total corrected fluorescence TDP-43 expression. Using confocal fluorescence microscopy, the percentage of transfected cells (visualised by the presence of EGFP fluorescence) bearing TDP-43 inclusions was quantified (Fig. 2A). Inclusions were defined as well delineated cytoplasmic structures visible using fluorescent microscopy. Inclusions were rarely formed (< 1%) in cells expressing wild-type TDP-43, but they were present in significantly more cells expressing TDP-43^M337V^ (10%, ****p < 0.0001). However, following co-expression of ERp57 with mutant TDP-43^M337V^, this proportion was significantly decreased to 2% (****p < 0.0001, Fig. 2B). Furthermore, there were no significant differences in inclusion formation between these cells and wild-type TDP-43 expressing populations. Hence, ERp57 is protective against the formation of misfolded mutant TDP-43^M337V^ inclusions in Neuro-2a cells. It was not possible to examine inclusion formation in cells expressing TDP-43^Q331K^ because unlike TDP-43^M337V^, this mutant did not readily form inclusions visible by microscopy.

### ERp57 Reduces the Size of Mutant TDP-43 Inclusions and Co-Localises with TDP-43 in Neuronal Cells

**Fig. 3:**
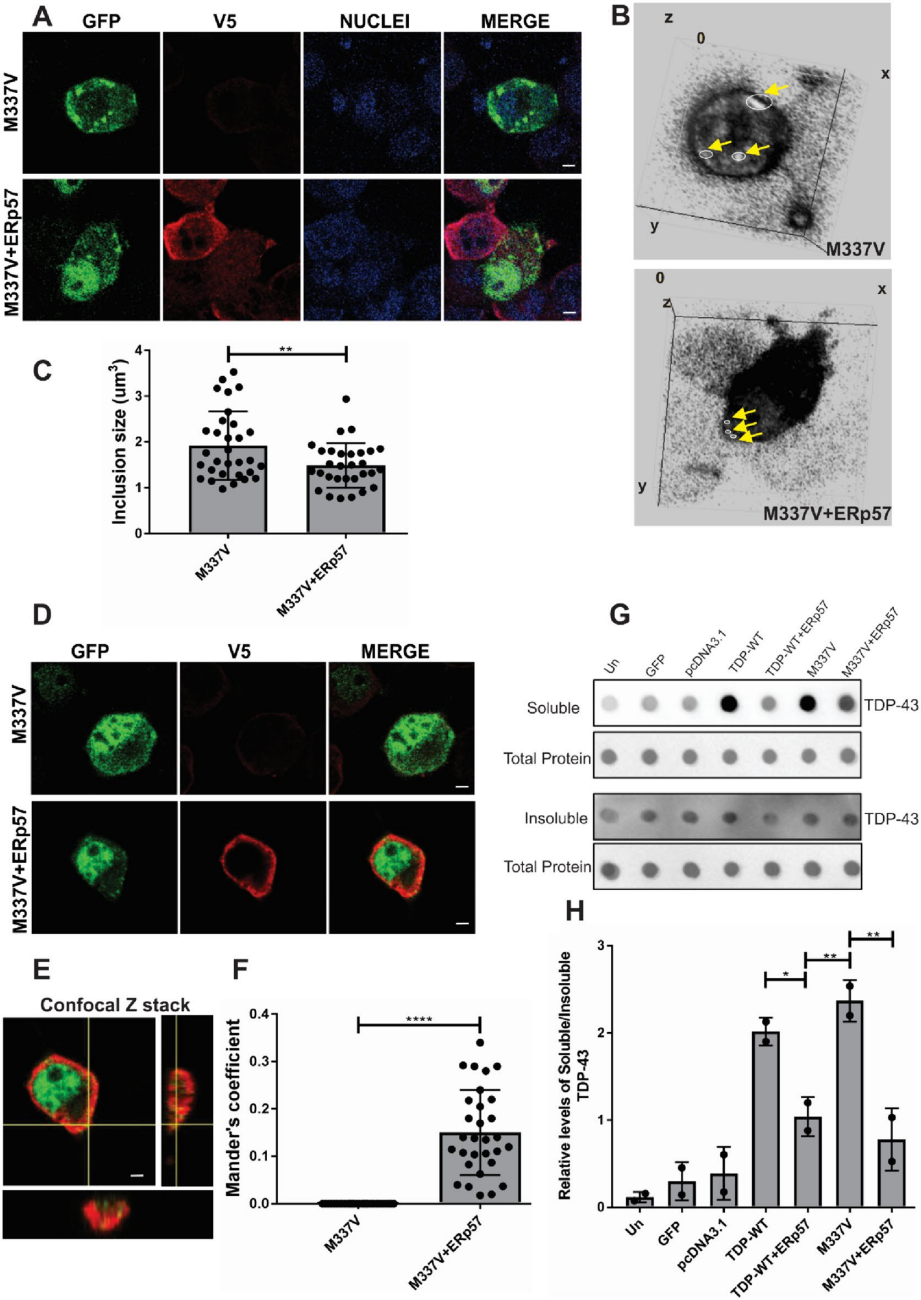
ERp57 decreases the size of mutant TDP-43^M337V^ inclusions and co-localises with TDP-43 in neuronal cell lines **A** Immunocytochemistry and confocal microscopy of GFP-positive inclusions in Neuro-2a cells expressing mutant TDP-43^M337V^ (M337V, green) with V5 tagged ERp57 (red), 72 h post transfection. Nuclei are visualised by Hoechst staining (blue). Scale bar = 5 µm**. B** 3D images using ImageJ depicting inclusion size variation shown with white circles and yellow arrows in ERp57 cells compared to TDP-43^M337V^ with vector alone. **C** Quantification using Z stack images (**A**) demonstrated that ERp57 significantly reduced inclusion size (**p < 0.01) compared to TDP-43^M337V^ with vector alone. **D** Immunofluorescence detection of GFP-positive TDP-43^M337V^ in Neuro-2a cells expressing empty vector (panel 1) with V5 tagged ERp57 (panel 2) demonstrated partially co-localized ERp57 with TDP-43-positive inclusions in neuronal cells. **E** Orthogonal section of the confocal image in (D), showing co-localization of TDP-43 inclusions with ERp57. **F** The degree of co-localization between TDP-43 inclusions with ERp57 in neuronal cells was quantified using Mander’s coefficient, revealing significant overlap (****p < 0.0001). Results are expressed as mean ± SD. **G** Protein lysates were subjected to vacuum filtration through a 96-well dot blot apparatus with a nitrocellulose membrane. Immunoblotting was then performed with anti-TDP-43 and anti-phospho-TDP-43 antibodies. Ponceau S staining was used as a reference for total protein levels (lane 2 and 4). Soluble TDP-43 levels (lane 1) and insoluble TDP-43 levels (lane 3) are shown. **H** Densitometric quantitation of TDP-43 protein (soluble/insoluble) levels normalized to total protein from the dot blot shown in (G) confirms that the ratio of soluble:insoluble wild-type or TDP-43^M337V^ was reduced in ERp57 expressing cells (**p < 0.01, *p < 0.05)

We also examined the size of the inclusions formed in each case to further characterise the effect of ERp57 on TDP-43 misfolding (Fig. 3A). Using confocal microscopy, Z stack images were acquired to provide three-dimensional images of TDP-43 inclusions (Fig. 3B). The size of these TDP-43 inclusions was then calculated (Fig. 3C). Mutant TDP-43^M337V^ formed inclusions with a mean size of 1.9 um^3^ in cells co-transfected with empty vector. However, cells co-expressing ERp57 displayed significantly smaller inclusions (mean size = 1.4 um^3^, **p < 0.01), implying that ERp57 inhibits the misfolding of TDP-43. We also detected partial co-localisation of ERp57 with TDP-43 (Fig. 3D, E). Mander’s coefficient was calculated in cells co-expressing ERp57 with TDP-43^M337V^ inclusions, where overlap between the two channels was represented between 1 (co-localised) and 0 (no co-localisation), which revealed that ERp57 co-localised with TDP-43 inclusions (Fig. 3F, ****p < 0.0001).

To further examine the effect of ERp57 on TDP-43 misfolding, a filter trap assay was performed to quantify TDP-43^M337V^ aggregation, using both soluble and insoluble lysates (Fig. 3G). However, to account for the differences in TDP-43^M337V^ expression between cells expressing ERp57 and empty vector, the ratio of insoluble:soluble protein (representing aggregated TDP-43) was compared, rather than the absolute levels of each fraction. TDP-43 aggregation was decreased in samples co-expressing ERp57 (Fig. 3H). Significant differences were observed (**p  <  0.01) in cells expressing mutant TDP-43^M337V^ and ERp57 compared to TDP-43^M337V^ with empty vector.

### ERp57 Does Not Affect Protein Unfolding Induced by Mutant TDP-43 in Neuronal Cells

**Fig. 4:**
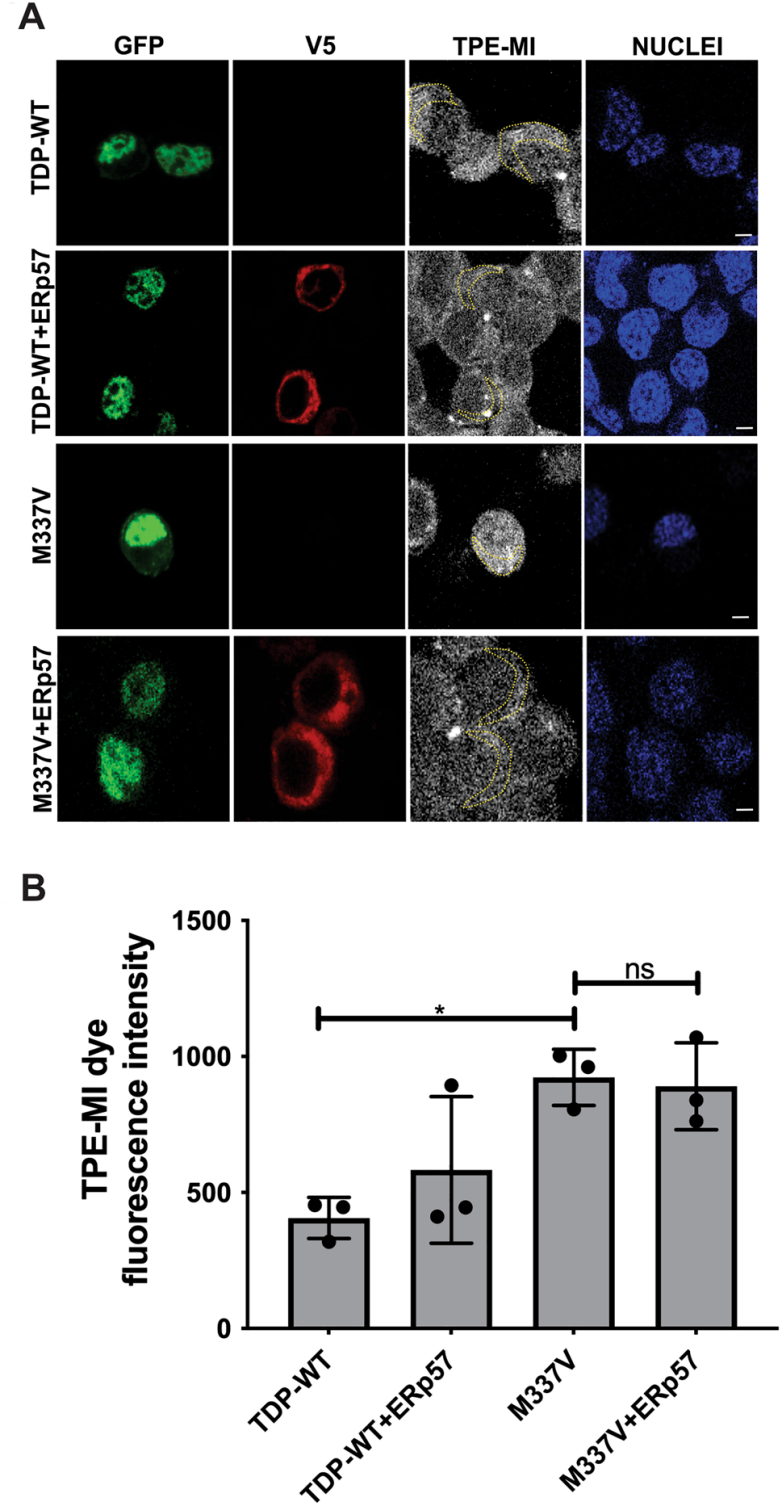
ERp57 has no effect on mutant TDP-43 unfolding in neuronal cell lines **A** Protein unfolding detection using TPE-MI  dye fluorescence in Neuro-2a cells. TDP-WT with empty vector (panel 1) and with V5 tagged ERp57 (panel 2), TDP-43^M337V^ with empty vector alone (panel 3), or co-expressing ERp57 (panel 4). Scale bar = 5 µm. Yellow dotted area representing TPE-MI levels in the cytoplasm. **B** TPE-MI fluorescence in the cytoplasm was significantly lower in cells expressing TDP-WT compared to TDP-43^M337V^ (*p < 0.05). However, no statistical difference was observed when ERp57 was co-expressed with TDP-43^M337V^ compared to controls, ns, non-significant

Unfolded proteins are highly susceptible to protein misfolding and aggregation, and we previously established that mutant TDP-43^M337V^ enhances protein unfolding using the fluorigenic dye tetraphenylethene maleimide (TPE-MI) in cells (Parakh et al., 2020). TPE-MI fluoresces upon recognition of free cysteine thiols normally buried in the folded state of globular protein domains, hence it can be used to monitor protein unfolding following treatment of cells for 30 min (M. Z. Chen et al., 2017). Next, we used TPE-MI to examine the load of unfolded proteins in cells expressing TDP-43 and ERp57 with steady-state levels (mid higher range > 3000 fluorescence intensity). Quantification revealed that significantly more TDP-43^M337V^ cells displayed protein unfolding, as previous (*p < 0.05) (Fig. 4A). However, no significant differences in fluorescence were detected in cells co-expressing TDP-43^M337V^ with ERp57, compared to empty vector (Fig. 4B). Hence, these data reveal that whilst ERp57 inhibits TDP-43^M337V^ inclusion formation and reduces their size, it has no effect on protein unfolding in neuronal cells.

### ERp57 Inhibits ER Stress Induced by Mutant TDP-43 in Neuronal Cells

**Fig. 5:**
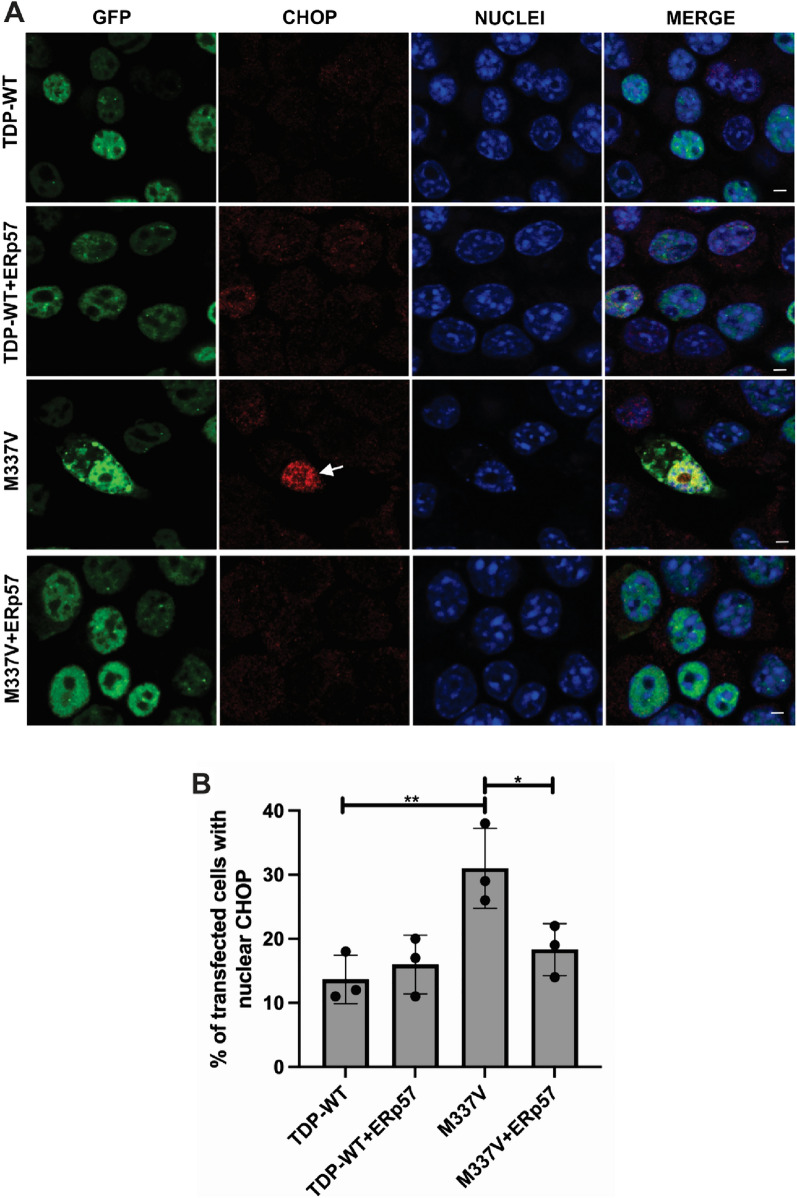
ERp57 inhibits ER stress induced by mutant TDP-43^M337V^ in neuronal cell lines **A** Immunocytochemistry and confocal microscopy of Neuro-2a cells expressing wild-type TDP-43 (TDP-WT) or mutant TDP-43^M337V^ (M337V, green) with V5 tagged ERp57, and nuclear CHOP immunoreactivity (red), were examined at 72 h post transfection. Nuclei were visualised by Hoechst staining (blue). A small proportion of cells expressing wild-type TDP-43 displayed nuclear CHOP (panel 1), and this was not altered by co-expression with ERp57 (panel 2). More nuclear CHOP immunoreactivity was present in populations expressing mutant TDP-43^M337V^ with vector alone (panel 3), represented by white arrows. In contrast, fewer cells formed inclusions when ERp57 was co-expressed with mutant TDP-43^M337V^ (panel 4). White arrows represent nuclear CHOP. Scale bar = 5 µm. **B** Quantification of the percentage of transfected Neuro-2a cells showing nuclear CHOP immunoreactivity in 5A. Results are expressed as mean ± SD, n = 3. Significant fewer cells expressing nuclear CHOP in wild-type TDP-43 compared to mutant TDP-43^M337V^ populations (**p < 0.01). Significantly fewer cells displayed nuclear CHOP when ERp57 was co-expressed with TDP-43^M337V^ (*p < 0.05) compared with cells transfected with pcDNA3.1 empty vector alone

Previously, it has been demonstrated that mutant TDP-43 induces ER stress in neuronal cells and activates CHOP, a pro-apoptotic, downstream UPR marker (X. Chen et al., 2023; Parakh et al., 2020; Walker et al., 2013). Therefore, the effect of ERp57 on ER stress in cells expressing mutant TDP-43^M337V^ was examined. Neuro-2a cells were co-transfected with TDP-43, with ERp57-V5 or empty vector. Nuclear immunoreactivity to CHOP at 72 h post transfection was used as a marker of ER stress, as previous (Fig. 5A). Following immunocytochemistry using anti-CHOP antibodies, in cells expressing wild-type TDP-43, a slight, but non-significant, activation of CHOP was detected in 14% of transfected cells. Similarly, co-expression of ERp57 with wild-type TDP-43 did not increase the proportion of cells with ER stress (16%). Consistent with our previous finding (Parakh et al., 2020), in cells expressing mutant TDP-43^M337V^, the proportion of transfected cells with CHOP activation increased significantly compared to those expressing wild-type TDP-43 (31%, **p < 0.01, Fig. 5B). Moreover, significantly fewer cells co-expressing ERp57 with TDP-43^M337V^ displayed nuclear CHOP immunoreactivity compared to those expressing empty vector (18%, *p < 0.05). Hence these results indicate that ERp57 is protective against ER stress induced by mutant TDP-43.

### ERp57 Inhibits Cell Death Induced by Mutant TDP-43^M337V^ in Neuronal Cell Lines

**Fig. 6:**
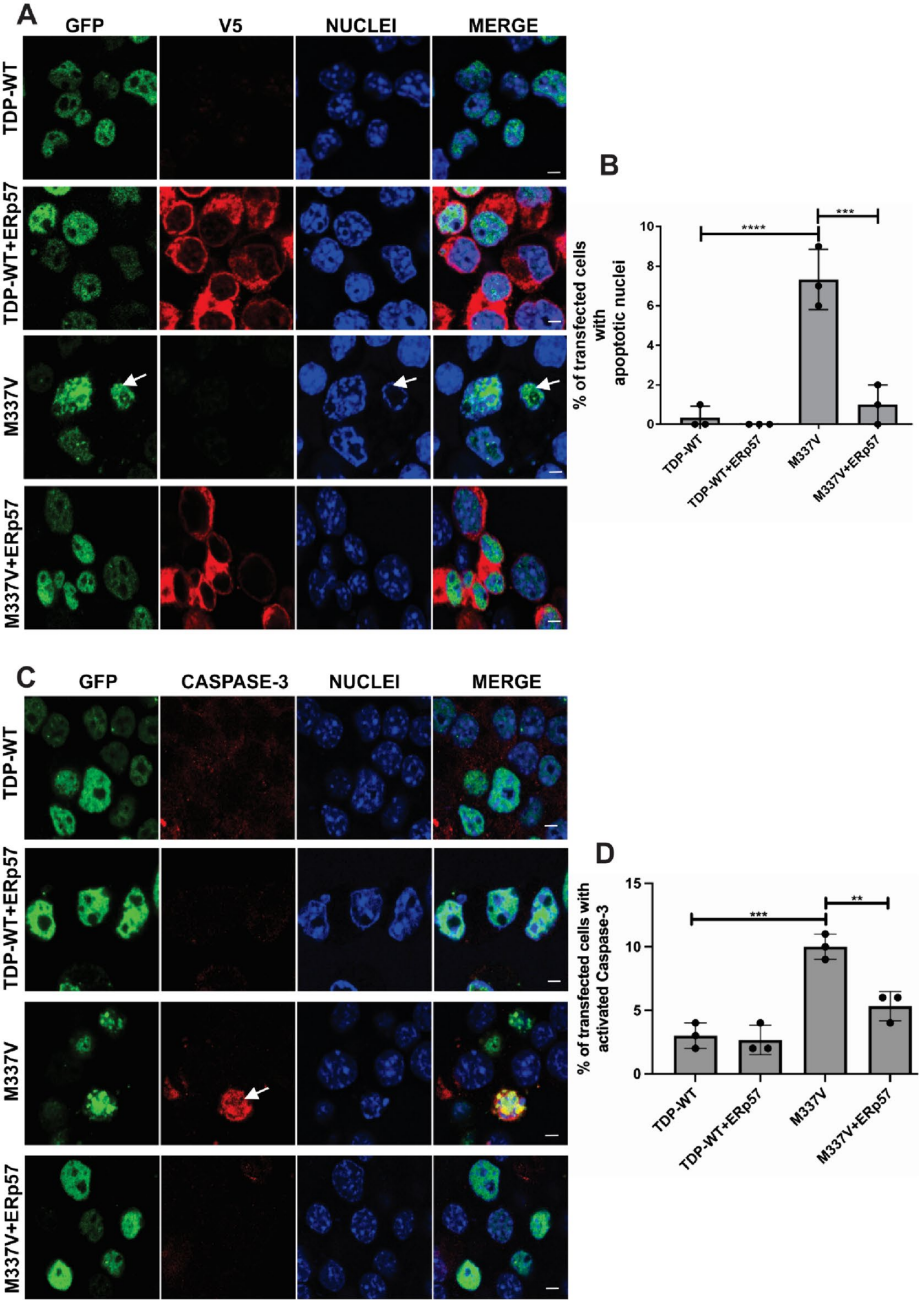
ERp57 protects against mutant TDP-43 induced cell death in neuronal cell lines **A** Neuro-2a cells were co-expressed with wild-type TDP-43 (TDP-WT) or TDP-43^M337V^ (M337V, green) and V5 tagged ERp57 (red), examined by confocal microscopy at 72 h post transfection. Nuclei are shown by Hoechst stain (blue). Arrow represents condensed or fragmented nuclei, indicating apoptosis is underway. Few cells expressing TDP-WT (panel 1) or TDP-WT co-expressing ERp57 (panel 2) contained fragmented nuclei and hence were apoptotic (< 1%) but more cells expressing TDP-43^M337V^ (panel 3) displayed Hoechst-stained condensed nuclei, indicating apoptosis, indicated by white arrows (middle panel). However, fewer cells co-expressing TDP-43^M337V^ with ERp57 (panel 3) were undergoing apoptosis compared to those transfected with empty vector, scale bar = 5 µm. **B** Quantification of apoptotic nuclei in cells in 5A expressing TDP-43 and ERp57. Results are expressed as mean ± SD, n = 3. A significant difference in apoptosis was observed between wild-type TDP-43 and TDP-43^M337V^ cells (****p < 0.0001). Over-expression of ERp57 with TDP-43^M337V^ resulted in significantly fewer cells undergoing apoptosis compared to cells transfected with empty vector only (***p < 0.001). **C** Activated caspase-3 immunoreactivity, confirming induction of apoptosis, in cells expressing TDP-43^M337V^ and ERp57. Neuro-2a cells were co-expressed with either wild-type TDP-43 or TDP-43^M337V^ (green) and ERp57 for 72 h, followed by immunocytochemistry using anti-activated caspase-3 antibodies (red), visualized using confocal microscopy. Nuclei are shown by Hoechst stain (blue). White arrow represents caspase-3 activation, indicating apoptosis is underway. As expected, fewer cells expressing wild-type TDP-43 (row 1) displayed caspase-3 activation, compared to cells expressing TDP-43^M337V^ (row 3). However, fewer cells expressing TDP-43^M337V^ with ERp57 (row 4) displayed caspase-3 activation, compared to those TDP-43^M337V^ cells transfected with empty vector. **D** Quantification of transfected cells visualized in 6C, immunostained using anti-activated caspase-3 antibodies. Results are expressed as mean ± SD, n = 3. Over-expression of ERp57 with TDP-43^M337V^ significantly decreased the proportion of cells with activated caspase-3, indicating apoptotic cell death is underway, compared to cells expressing empty vector only (**p < 0.01, ***p<0.001 )

Mutant TDP-43^M337V^ triggers apoptosis in neuronal cells (Parakh et al., 2020; Vogt et al., 2018). Hence it was next examined whether ERp57 inhibits apoptosis in cells expressing mutant TDP-43^M337V^ by quantifying the proportion of cells with fragmented or condensed nuclei, a recognised indicator of apoptosis (Dmitrieva & Burg, 2008; Mandelkow et al., 2017; Parakh et al., 2021; Toné et al., 2007; A. K. Walker et al., 2010), as previous, (Parakh et al., 2020). Cells expressing wild-type or mutant TDP-43^M337V^ were examined for the presence of apoptotic nuclei (Fig. 6A), following counter-staining for Hoechst, at 72 h post transfection. Similar to previous findings (Parakh et al., 2020), cells expressing wild-type or mutant TDP-43^M337V^ that did not form inclusions displayed normal nuclear morphologies, indicating negligible levels of apoptotic cell death. In contrast, 7% of TDP-43^M337V^ expressing cells that bore inclusions contained fragmented nuclei, demonstrating that apoptosis was underway (****p < 0.0001). However, when ERp57 was co-expressed with mutant TDP-43^M337V^, significantly fewer cells were undergoing apoptosis compared to cells expressing TDP-43^M337V^ alone: only 1% displayed condensed or fragmented nuclei (***p < 0.001, Fig. 6B). Hence, these results imply that ERp57 is protective against apoptosis triggered by mutant TDP-43^M337V^.

To further validate the above findings, we examined the presence of the activated, cleaved form of caspase-3 in cells co-expressing ERp57 with mutant TDP-43 as a specific marker of apoptosis. Neuronal cells were examined 72h post-transfection and similar to the above results, wild-type TDP-43 (3%) cells and cells co-expressing ERP57 and wild-type TDP-43 (3%) displayed little caspase-3 activation, indicating negligible levels of apoptotic cell death (Fig. 6C). In contrast, significantly more cells (10%, ***p < 0.001,) expressing TDP-43^M337V^ displayed activated caspase-3, demonstrating that apoptosis was underway in these populations. However, when ERp57 was co-expressed with mutant TDP-43^M337V^, significantly fewer cells (5%, **p < 0.01, Fig. 6D) displayed caspase-3 activation compared to those expressing mutant TDP-43^M337V^ with empty vector, confirming that ERp57 is protective against apoptosis.

Together, these results therefore demonstrate that ERp57 co-localises with mutant TDP-43 inclusions and is protective against TDP-43 misfolding, inclusion formation, and apoptosis, in cells expressing mutant TDP-43.

## Discussion

In this study, we identify novel protective roles for ERp57 against pathological forms of TDP-43, which are associated with most ALS cases (97%) and ~ a significant proportion of FTD and Alzheimer’s’ disease cases. We showed that ERp57 inhibits the mislocalisation of TDP-43^M337V^ to the cytoplasm and the formation of inclusions, two key features of TDP-43 pathology. We also detected smaller TDP-43^M337V^ inclusions in cells expressing ERp57, further implying that ERp57 inhibits TDP-43 misfolding, which we confirmed using the filter-trap assay. In addition, we showed that ER57 is protective against ER stress and apoptosis in mutant TDP-43^M337V^ expressing cells. Our results therefore imply that TDP-43 is a possible client of the ERp57-folding pathway, facilitating the correct folding of misfolded TDP-43. Consistent with this notion, we also demonstrated that ERp57 co-localises with TDP-43 inclusions in neuronal cells, in accordance with our previous findings whereby ERp57 co-localised with TDP-43 inclusions in sporadic ALS patients (Parakh et al., 2018).

Misfolding of TDP-43 induces conformational changes and its subsequent aggregation into inclusions, a pathological hallmark of ALS. ERp57 is a thiol isomerase that catalyses protein oxidation, reduction and disulphide isomerization reactions during protein folding, which mediates disulphide bond formation (Frickel et al., 2004). ERp57, like PDI, can prevent misfolding and it is increasingly implicated in neurodegenerative diseases (Bilches Medinas et al., 2022). The close proximity and availability of ERp57 to TDP-43, as detected here and in our previous study (Parakh et al., 2018), may therefore enable ERp57 to solubilize misfolded aggregates and thus inhibit the formation of pathological TDP-43.

However, here ERp57 was not protective against protein unfolding in TDP-43 ^M337V^ expressing cells. This contrasts with our previous findings (Parakh et al., 2020) whereby PDI did alleviate mutant TDP-43^M337V^-induced protein unfolding, also detected using TPE-MI dye (M. Z. Chen et al., 2017). These findings imply that PDI and ERp57 may operate on distinct conformational populations of unfolded and misfolded TDP-43. This implies that there are subtle differences between ERp57 and PDI in their protective functions in ALS. Consistent with this notion, PDI, but not ERp57, functions in the ER-associated degradation pathway by which misfolded ER proteins are retro-translocated to the cytoplasm for ubiquitination and degradation by the proteasome (Zhao et al., 2021). Whilst both ERp57 and PDI share the same domain architecture (a-b-b’-a’-c) and are the most closely related members of the PDI family (Maattanen et al., 2006), ERp57 is also distinct from PDI because of its different substrate specificities and redox potential. Another possibility to account for the differences between PDI and ERp57 in protein unfolding may relate to the preference of ERp57 for heavily glycosylated substrates (Catherine E Jessop et al., 2007) because ERp57, unlike PDI, is involved in the folding of glycoproteins as part of the calnexin/calreticulin cycle (Zhang et al., 2009). Hence the effectiveness of ERp57 in ALS may be dependent on collaboration with co-chaperones calnexin and calreticulin because these proteins function together (Saibil, 2013).

A recent study showed that TDP-43 can be O‐glycosylated (Zhao et al., 2021). Importantly, this modification suppressed features associated with TDP-43 pathology, TDP‐43 aggregation and hyperphosphorylation, and also promoted its splicing functions, including splicing of STMN2 mRNA, which is required for axonal outgrowth (Zhao et al., 2021). Hence, it is possible that ERp57 specifically recognises and promotes the folding of this O‐glycosylated form of TDP-43, rather than its unfolded form, which may account for some of the differences we detected between PDI and ERp57.

Interestingly, we also observed differences in the steady-state levels of TDP-43 in this study, but only at lower expression levels (< 3000 total corrected fluorescence). In contrast, we did not previously detect alterations in expression of TDP-43 in the presence of PDI (Parakh et al., 2020). Hence, this also implies that there are differences in the effect of PDI and ERp57 on expression of their substrate proteins. ERp57 has been previously shown to regulate the steady-state levels of wild-type and mutant prion protein (PrP), up to 50% (Torres et al., 2015), implying that it can modulate the expression of proteins closely associated with neurodegeneration. Similarly, PDI is also known to regulate the translation of insulin granule proteins by binding to 5’ UTR sequences within their mRNA (Sarwade et al., 2020). TDP-43 regulates its own expression levels by a negative feedback loop in which it targets a sequence in the 3′ UTR of its own transcript (Ayala et al. 2011). This can be triggered by elevated levels of TDP-43 protein which induces nonsense-mediated decay of its mRNA (Polymenidou et al., 2011). Thus, inhibition of TDP-43 expression by ERp57 may involve this autoregulatory process.

Although it is considered to be primarily resident in the ER, ERp57 has also been detected in the nucleus, mitochondria and cytosol (Turano et al., 2002). ERp57 can undergo significant dynamic changes in conformation and cellular localization in response to cellular conditions, and ER stress can trigger re-distribution of ERp57 into the cytoplasm (Chichiarelli et al., 2022). In this study, we also found that ERp57 expression inhibited mutant TDP-43 (M337V and Q331K) mislocalisation in the cytoplasm in neuronal cells, using both mCherry and GFP tagged proteins. Together, these findings point to the cytoplasm as the cellular compartment where ERp57 and TDP-43 actively interact.

Non-native disulphide bonds are implicated in the aggregation of mutant TDP-43 (Cohen et al., 2012). Moreover wild-type TDP-43 was previously shown to form oligomers and aggregates under pathological conditions (Jiang et al., 2017). Consistent with these findings, we detected wild-type TDP-43 aggregates in the filter trap assay, although these probably small oligomers rather than the inclusions visible by microscopy, because few wild-type TDP-43 inclusions were detected in this study. We previously showed that the thiol activity of PDI mediates its protective role against mutant TDP-43^M337V.^ Hence, it is possible that the thiol activity of ERp57 is also responsible for its protective function against pathological TDP-43, although further studies are required to confirm this. We also show that ERp57 is protective against ER stress (detected by CHOP immunoreactivity) and apoptosis induced by mutant TDP-43^M337V^ using two assays; DAPI condensation and caspase-3 activation. CHOP is a pro-apoptotic marker, and ERp57 inhibited CHOP activation, implying that it is protective in the late phases of UPR. The protective effect of ERp57 on neuronal apoptosis implies that it has potential to prevent the death of motor neurons, which is central to ALS. Hence, these findings imply that delivering ERp57 therapeutically to enhance the clearance of TDP-43 pathology and prevent neuronal death may be beneficial given that endogenous ERp57 could be compromised due to protein oxidation and aggregation during ALS. Recently, ERp57 over-expression in transgenic SOD1^G93A^ mice reduced neuromuscular decline and SOD1 aggregation at late disease stages, revealing that ERp57 is protective in vivo in ALS, although this mouse model does not display the TDP-43 pathology characteristic of most cases (Rozas et al., 2021).

It is important to mention possible caveats to this study. Cell lines overexpressing ALS-associated mutant TDP-43 were used here to gain a simplified, yet accelerated, view of the pathological consequences of expression of mutant TDP-43. However, transient transfections result in high levels of protein expression, which may be non-physiological. Investigations using stable cell lines may eliminate potentially aberrant levels of protein expression. Also this study did not examine the protective effect of ERp57 in vivo, which could be investigated in the future. Although beyond the scope of the current study, it would be important in the future to define the exact mechanisms by which ERp57 regulates TDP-43 expression, and whether this is at the mRNA or protein level. It also remains unclear in which cellular compartment TDP-43 and ERp57 interact and thus from where ERp57 is protective in ALS. This is likely to be the cytoplasm, but further studies are required to confirm this.

In summary, here we demonstrate that a novel PDI-family member, ERp57, is protective against key features associated with TDP-43 pathology, the major hallmark of ALS, that is also present in several other neurodegenerative conditions, including FTD and AD. This study, therefore, provides new insights into developing novel therapeutics for neurodegenerative disorders based on the broad function of PDI family members in ALS.

## Supplementary Information

Below is the link to the electronic supplementary material.

Supplementary file1 (TIF 52562 KB)

Supplementary file2 (TIF 29509 KB)

Supplementary file3 (PDF 247 KB)

## Data Availability

The datasets used and/or analyzed during the current study are available from the lead author on reasonable request.
