# Supplementary material for: Protein Disulfide Isomerase Endoplasmic Reticulum Protein 57 (ERp57) is Protective Against ALS-Associated Mutant TDP-43 in Neuronal Cells
Source: Neuromolecular Med. 2024 Jun 11;26(1):23. doi: 10.1007/s12017-024-08787-0 (PMC11166824; doi:10.1007/s12017-024-08787-0)
Supplement: Supplementary file 3 — Supplementary file3 (PDF 247 KB) [file 12017_2024_8787_MOESM3_ESM.pdf]

Supplementary data

Methods

Cell lysates were collected by adding chilled Tris-NaCl buffer (50 mM Tris-HCl pH 7.5 and 150 mM NaCl, pH 7.6) with 0.1% [w/v] sodium dodecyl sulfate (SDS), 1% Triton-X100, 1% protease inhibitor cocktail (Roche) and 1% phosphatase inhibitor (Roche) following incubation on ice for 20 min. Lysates were stored at -20°C overnight. To obtain the SDS-soluble fraction, samples were centrifuged at 100,000 g at 4°C for 30 min. A BCA protein assay (ThermoFisher Scientific) was used to compare the protein concentrations of cell lysates to BSA standards. 20μg protein samples were electrophoresed on 8.5% SDS–polyacrylamide gels before being transferred to nitrocellulose membranes. Membranes were treated with the relevant primary antibodies: anti-TDP-43 (1:1000, ProteinTech, 10782-2-AP), anti-ERp57 (1:1000, Abcam ab13506), or anti-β-actin (1:1000, Sigma A2228) antibodies at 4°C for 24 hr, after blocking with 5% skim milk in Tris-buffered saline for 1 hr. Secondary antibodies (1:2000, HRP-conjugated goat anti-rabbit or goat anti-mouse, Merck Milipore, AP132, AP130) were incubated with membranes for 1 hr at room temperature before detection with ECL reagent (Bio-Rad). Dual-Color Precision Plus Protein Standard molecular weight markers was used (Bio-Rad).

Supplementary data

S1A

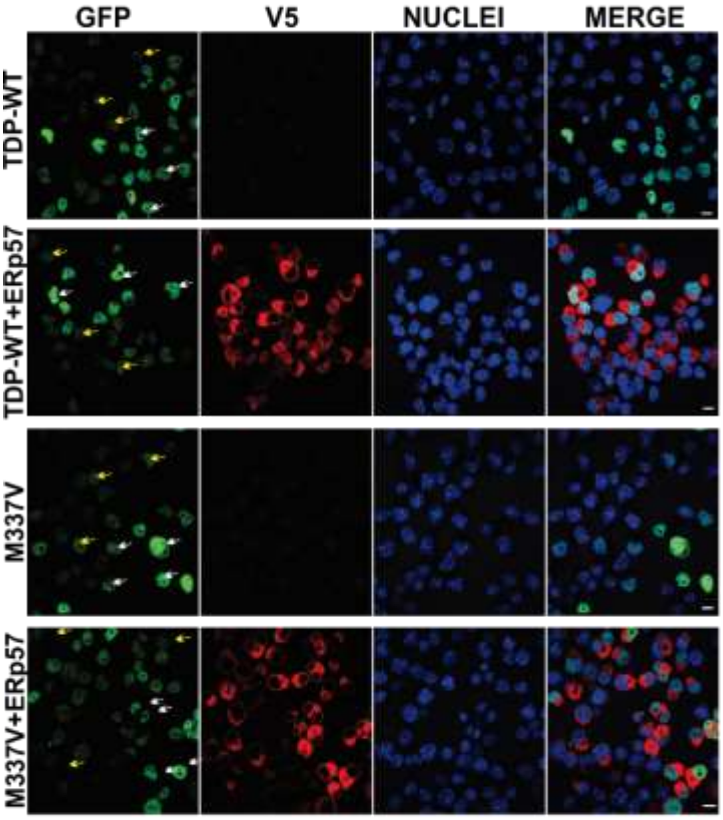

S1B

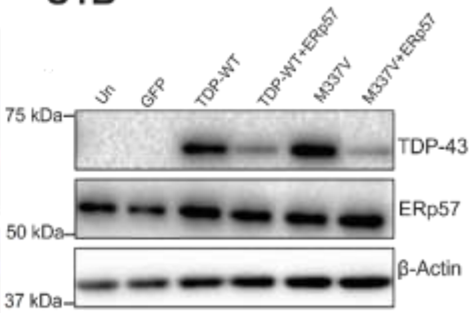

S1C

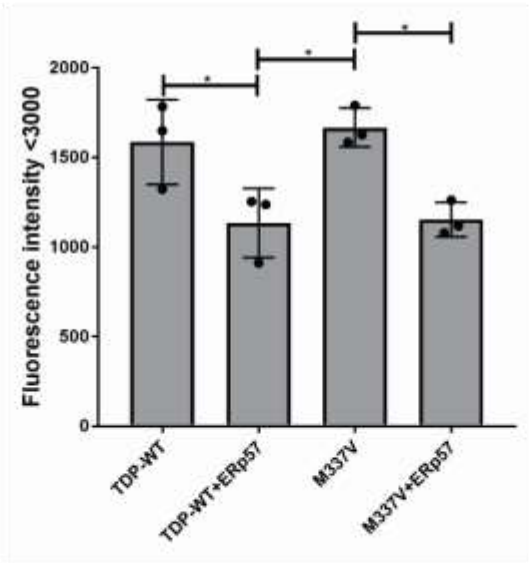

S1D

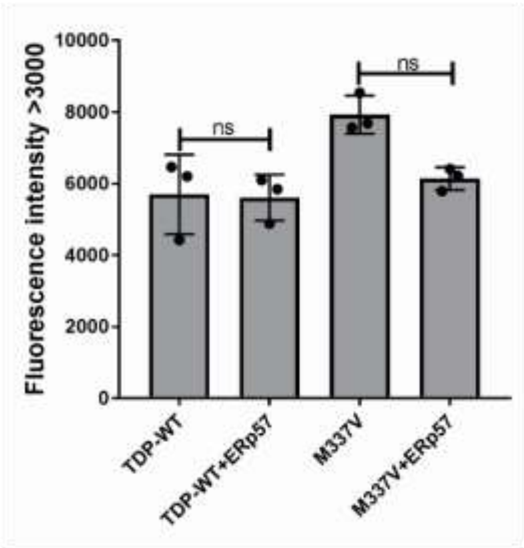

Figure legends

S1 Co-expression of ERp57 and TDP-43 in Neuro2a cells

A) Immunofluorescence detection of GFP-tagged TDP-43 and V5-tagged ERp57 in Neuro-2a cells expressing wild-type TDP-43 (TDP-WT) or mutant TDP-43^{M337V} (M337V, green) with V5 tagged ERp57 (red), examined by confocal microscopy, 72 hr post transfection. Nuclei are visualised by Hoechst staining (blue). 99% of cells expressing wild-type TDP-43 or mutant TDP-43^{M337V} also co-express ERp57. However, low (<3000 pixel intensity, yellow arrows) and high (>3000 pixel intensity, white arrows) intensity fluorescent cells were observed in both wild-type and mutant TDP-43 ^{M337V} expressing populations. Scale bar = 25 µm. **B)** Western blotting of Neuro-2a cell lysates, in which either wild-type TDP-43 or mutant TDP-43^{M337V} was co-expressed with either empty vector pcDNA3.1 or ERp57-V5, untransfected (Un) cells, empty vector GFP only. The blots were probed with anti-TDP-43 antibody (lane 1) and re-probed with anti-ERp57 antibody (lane 2) to confirm the presence of ERp57, with anti-β-actin as a loading control (lane 3). Approximate molecular weight markers in kilodaltons (kDa) are shown on the left. **C)** Quantification of expression levels of TDP-43 cells in Neuro-2a cells expressing low fluorescent intensity represented in (S1A). Results are expressed as mean ± SD, n=3. A significant difference in the expression levels of both wildtype and mutant TDP-43 in the presence or absence of ERp57 was detected (*$p<0.05$). **D)** Quantification of expression levels of TDP-43 cells in Neuro-2a cells expressing mid-high fluorescent intensity represented in (S1A). Results are expressed as mean ± SD, n=3. No significant diffference was observed in the expression levels of TDP-43 expressing cells with or without ERp57.

S2A

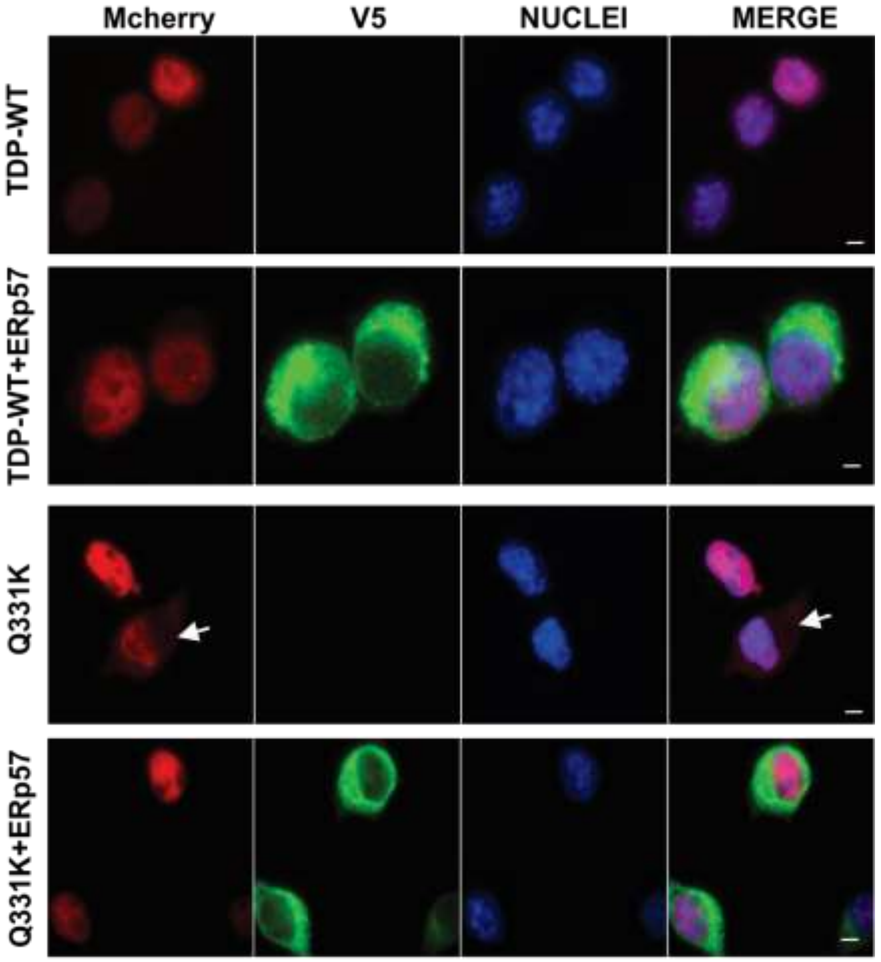
Mcherry
V5
NUCLEI
MERGE
TDP-WT
TDP-WT+ERp57
Q331K
Q331K+ERp57

S2B

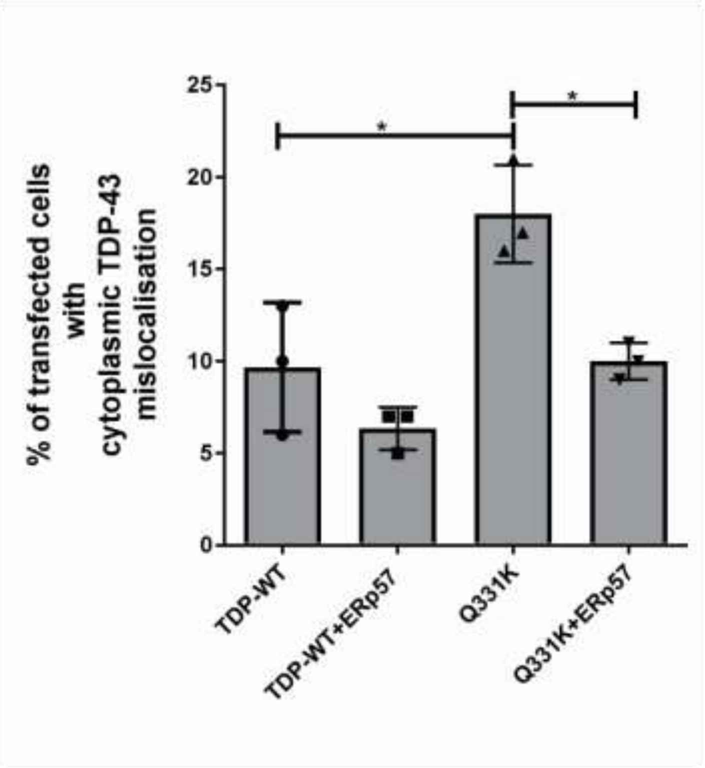
% of transfected cells with cytoplasmic TDP-43 mislocalisation
25
20
15
10
5
0
*
*
TDP-WT
TDP-WT+ERp57
Q331K
Q331K+ERp57

S2 ERp57 protects against mislocalisation of mutant TDP-43 mCherry from the nucleus to the cytoplasm in Neuro2a cells

A) Cells expressing mCherry tagged wild-type TDP-43 (TDP-WT, panel 1) or wild-type TDP-43 co-expressing ERp57 (TDP-WT+ERp57, panel 2) or empty vector displayed mainly nuclear TDP-43 localisation, whereas more cells expressing mutant mCherry TDP-43^{Q331K} (Q331K) exhibited cytoplasmic localisation, indicated with white arrow (panel 3). On co-expressing ERp57 with mutant TDP-43^{Q331K}, fewer cells displayed cytoplasmic TDP-43 localisation compared to those expressing TDP-43^{Q331K} with vector alone (panel 4) determined by confocal microscopy, at 18 hr post-transfection. Scale bar = 5 µm. **B)** Quantification of cells visualised in (S2A), displaying cytoplasmic distribution of mutant TDP-43. Results are expressed as mean ± SD, n=3. Over-expression of ERp57 with mutant TDP-43^{Q331K} significantly decreased the proportion of cells displaying cytoplasmic TDP-43, compared to TDP-43^{Q331K} cells expressing empty vector only (*$p<0.05$).
